# Supplementary material for: Surfactant-free interfacial growth of graphdiyne hollow microspheres and the mechanistic origin of their SERS activity
Source: Nat Commun. 2023 Oct 9;14:6318. doi: 10.1038/s41467-023-42038-3 (PMC10562396; doi:10.1038/s41467-023-42038-3)
Supplement: Supplementary file 1 — Supplementary Information [file 41467_2023_42038_MOESM1_ESM.pdf]

Supplementary Information

Surfactant-free interfacial growth of graphdiyne hollow microspheres and the mechanistic origin of their SERS activity

Lu Zhang,[1] Wencai Yi,[2] Junfang Li,[1] Guoying Wei,[3] Guangcheng Xi,*[1] and Lanqun Mao*[4]

[1] Key Laboratory of Consumer Product Quality Safety Inspection and Risk Assessment for State Market Regulation, Chinese Academy of Inspection and Quarantine, Beijing 100176 (China).

[2] School of Chemistry, Beijing Normal University, Beijing 100875 (China).

[3] School of Physics and Physical Engineering, Qufu Normal University, Qufu 273165 (China)

[4] School of Materials and Chemistry, China Jiliang University, Hangzhou 310018 (China)

*E-mail: xiguangcheng@caiq.org.cn, lqmao@bnu.edu.cn.

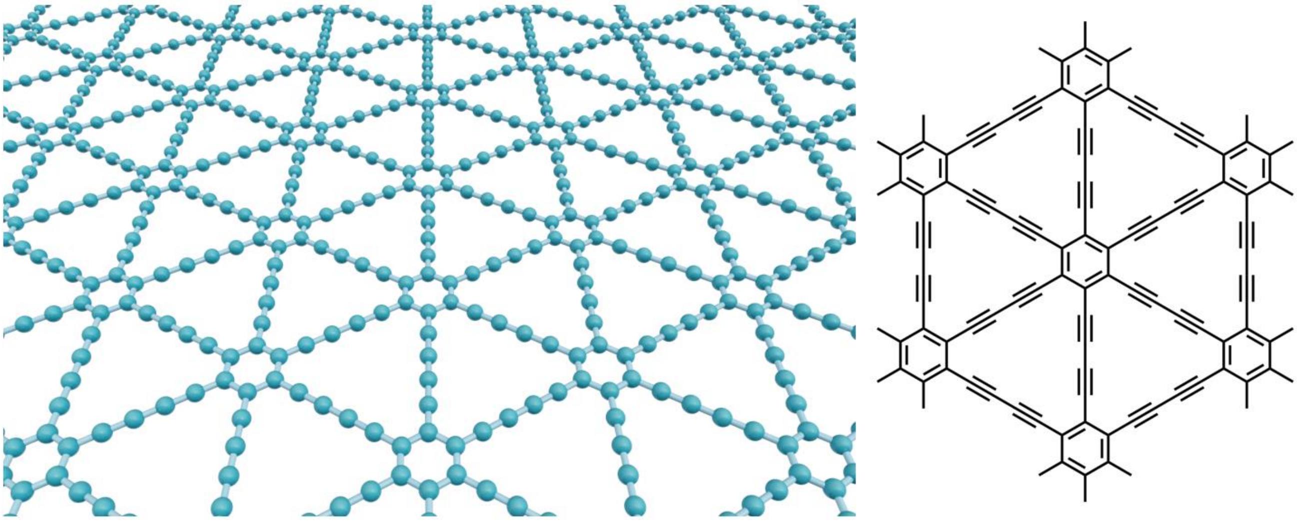

Supplementary Figure 1. Structure diagram of GDY. GDY has a unique 2D planar network structure, which is formed by inserting diacetylenic linkages between two benzene rings in the graphene structure.

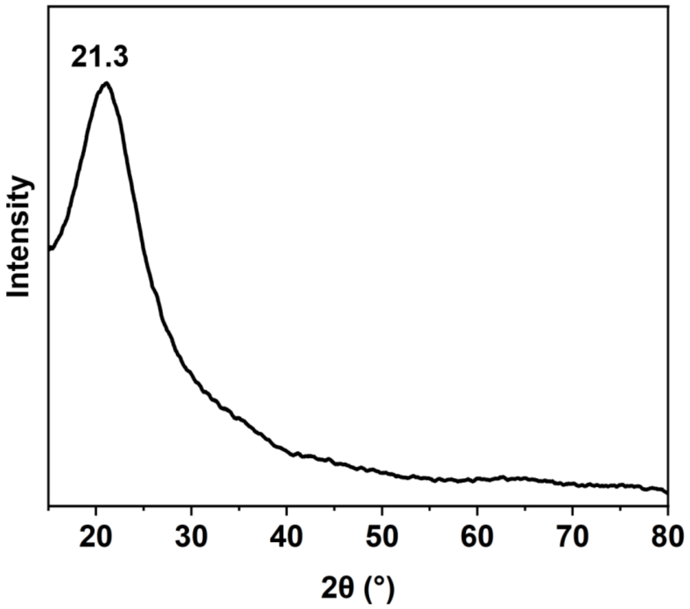

Supplementary Figure 2. XRD pattern of the as-synthesized GDY HHMSs. The strong diffraction peak at 21.3° shows the high crystallinity of these GDY products. Source data are provided as a Source Data file.

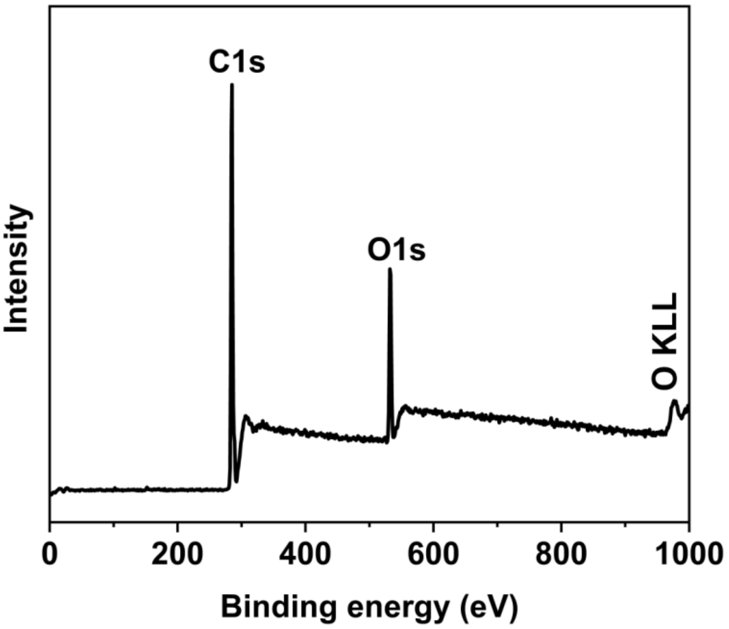

Supplementary Figure 3. XPS survey spectrum of the GDY HHMSs. Source data are provided as a Source Data file.

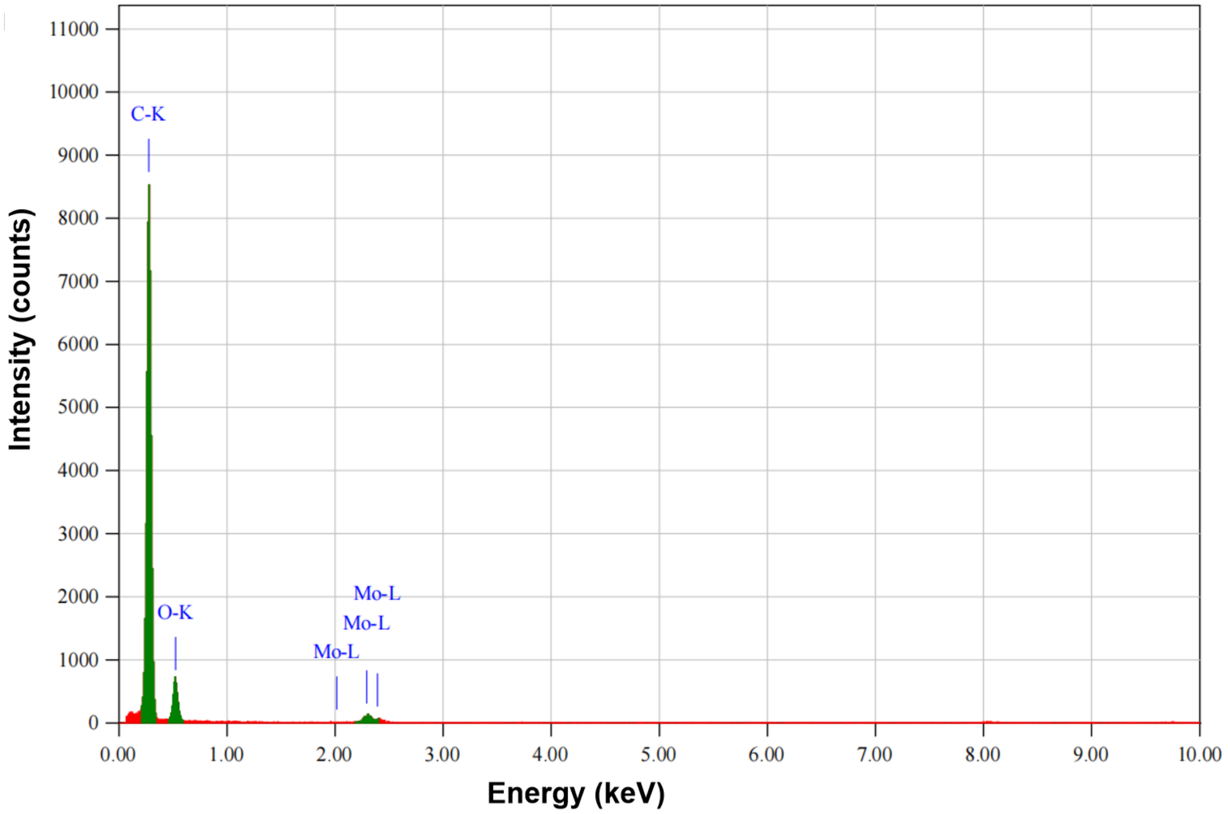

Supplementary Figure 4. EDS spectrum of the GDY HHMSs. Molybdenum signal comes from molybdenum grid. Source data are provided as a Source Data file.

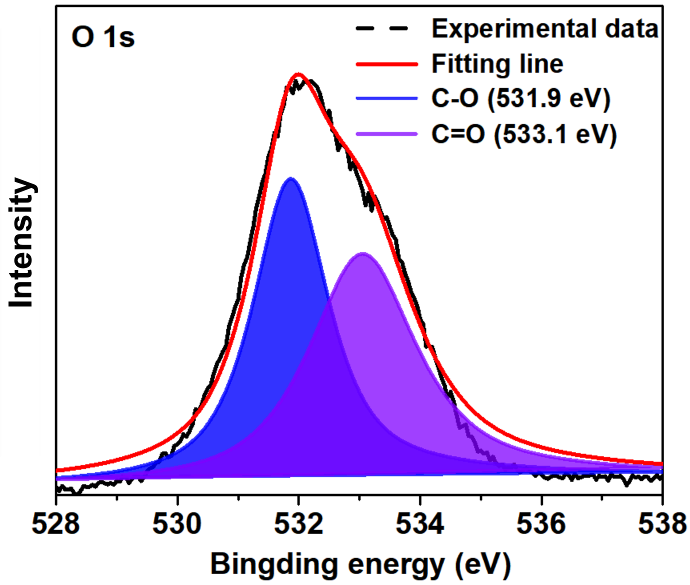

Supplementary Figure 5. O 1s spectrum of the GDY HHMSs. Source data are provided as a Source Data file.

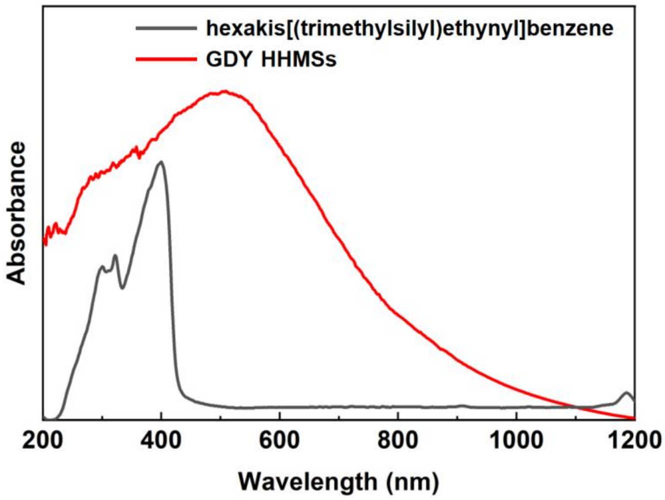

Supplementary Figure 6. UV-Vis absorbance spectra of GDY HHMSs and hexakis[(trimethylsilyl)ethynyl]benzene. Source data are provided as a Source Data file.

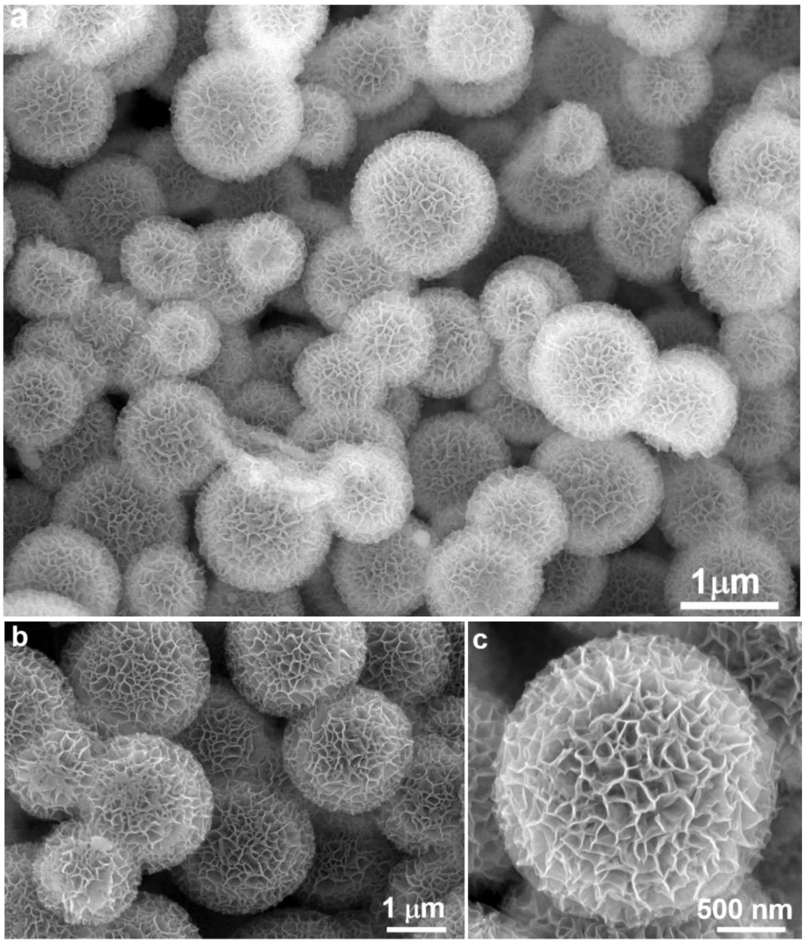

Supplementary Figure 7. SEM images of the as-synthesized GDY HHMSs. (a-c) SEM images with different magnifications.

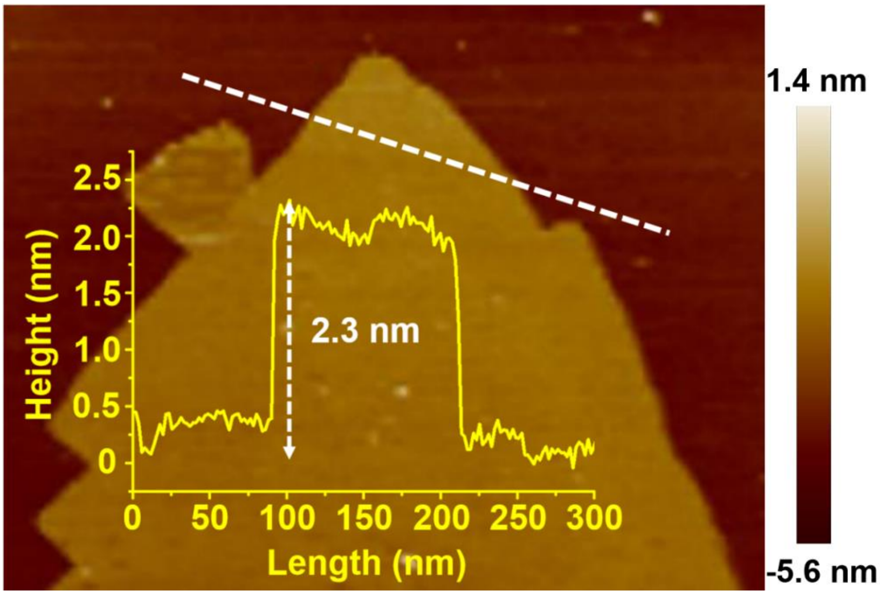

Supplementary Figure 8. AFM image of a single ultrathin nanosheet from the GDY HHMSs.

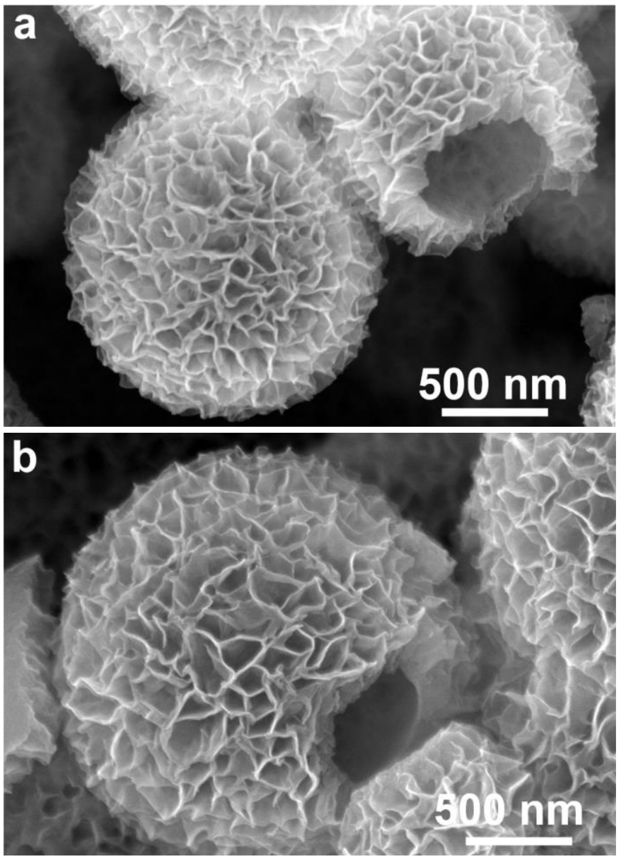

Supplementary Figure 9. (a,b) SEM images of broken GDY microspheres reveal that the interior of these spheres is hollow.

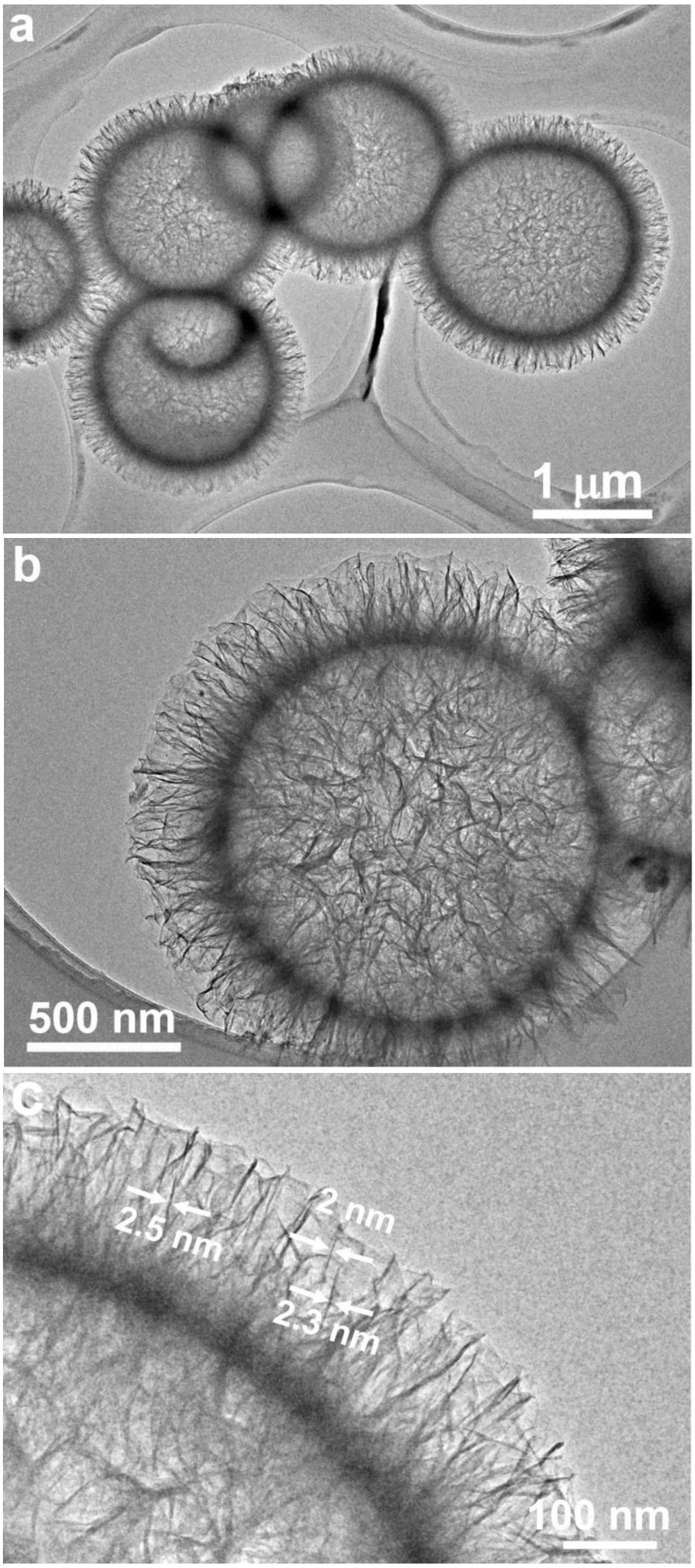

Supplementary Figure 10. TEM images of the GDY HHMSs. (a-b) TEM images with different magnifications. From these images, it can be clearly seen that these GDY products are composed of a large number of hollow microspheres assembled from ultrathin nanosheets. (c) Thickness measurement by the cross-section of the nanosheets.

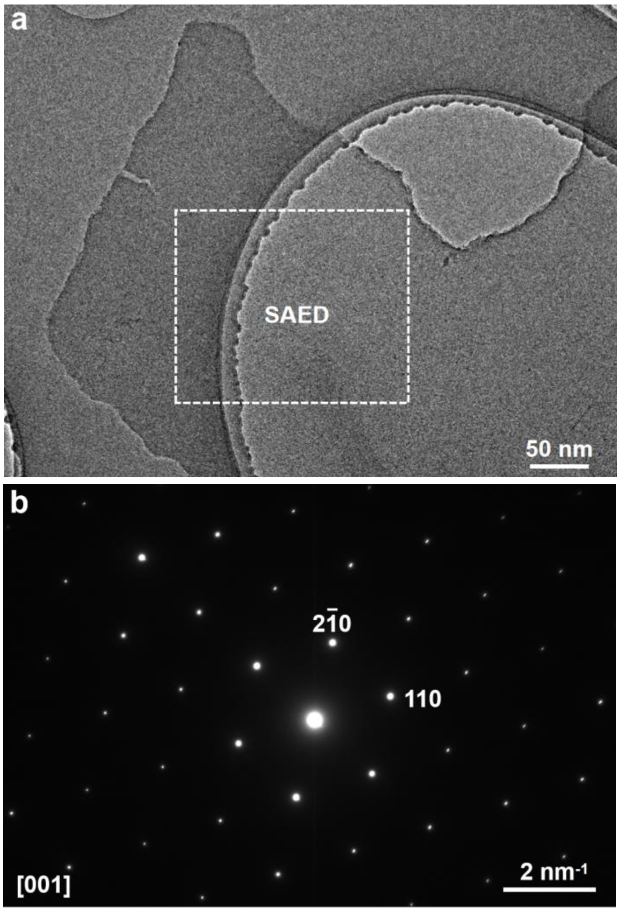

Supplementary Figure 11. High-magnification TEM image and SAED pattern of the GDY HHMSs. (a) TEM image and (b) corresponding SAED pattern of a single nanosheet of the GDY HHMSs. From the images, it can be clearly seen that these GDY products are highly crystalline.

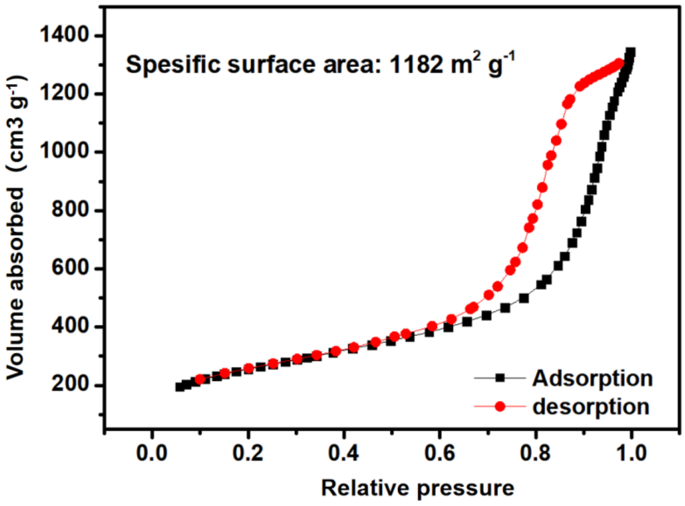

Supplementary Figure 12. N_2 adsorption and desorption isotherms of GDY HHMSs, which shows that its specific surface area is as high as 1182 m^2 g^{-1}. Source data are provided as a Source Data file.

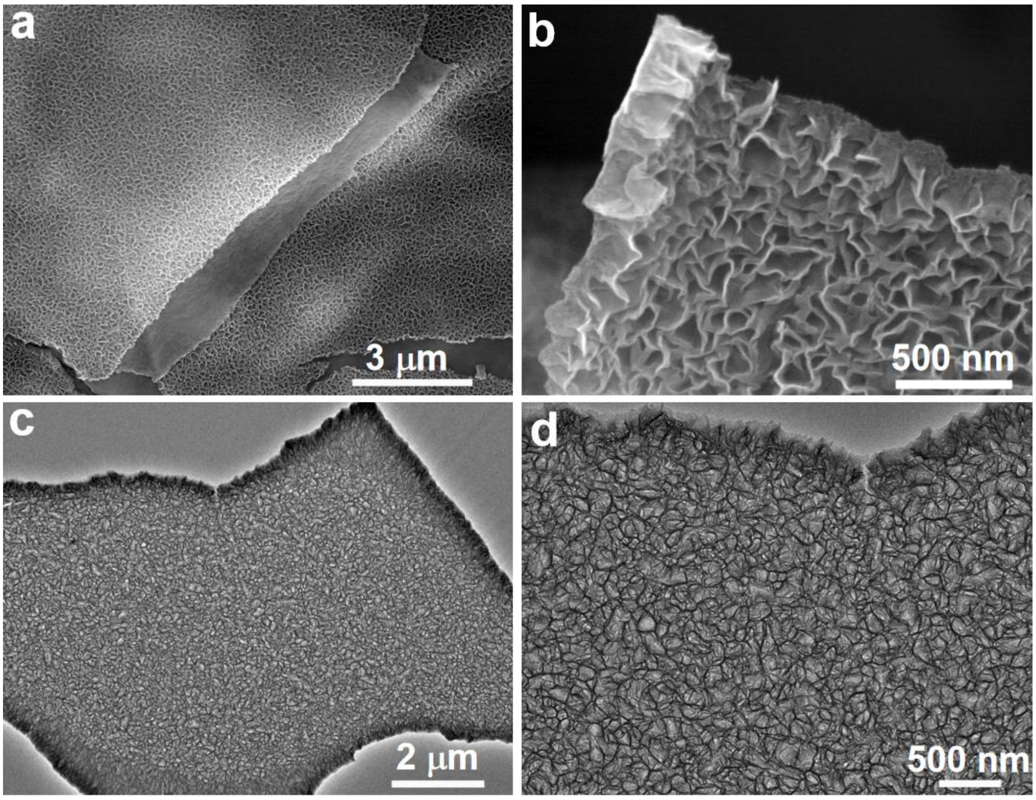

Supplementary Figure 13. Morphology and structure characterizations of the GDY HNPs. (a-b) SEM image, (c-d) TEM images. From these images, it can be clearly seen that these GDY HNPs are composed of a large number of nanosheets vertically arranged and assembled.

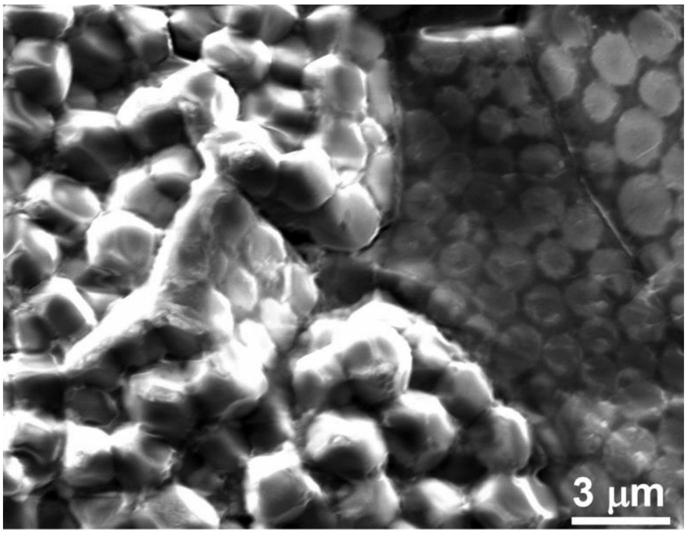

Supplementary Figure 14. The cryoelectron microscopy image of these spherical microemulsion droplets in the two-phase interface.

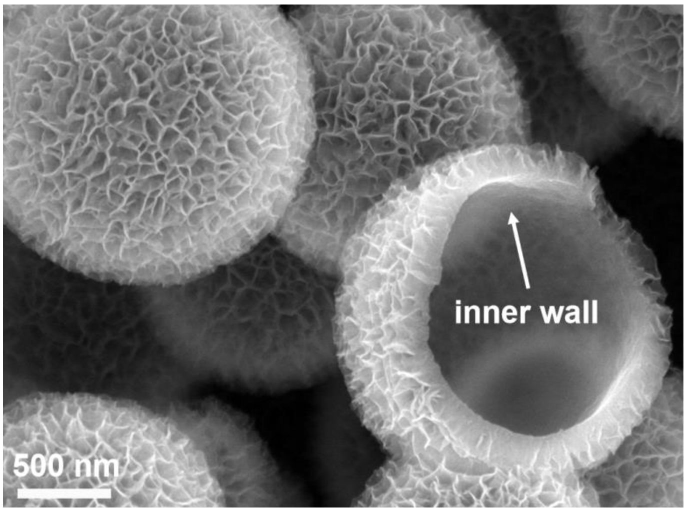

Supplementary Figure 15. SEM image of the GDY HHMSs. It can be seen from the photos that the inner and outer surfaces of these hollow microspheres are completely different. Specifically, the outer surface is a rough surface composed of nanosheets, while the inner surface is relatively smooth.

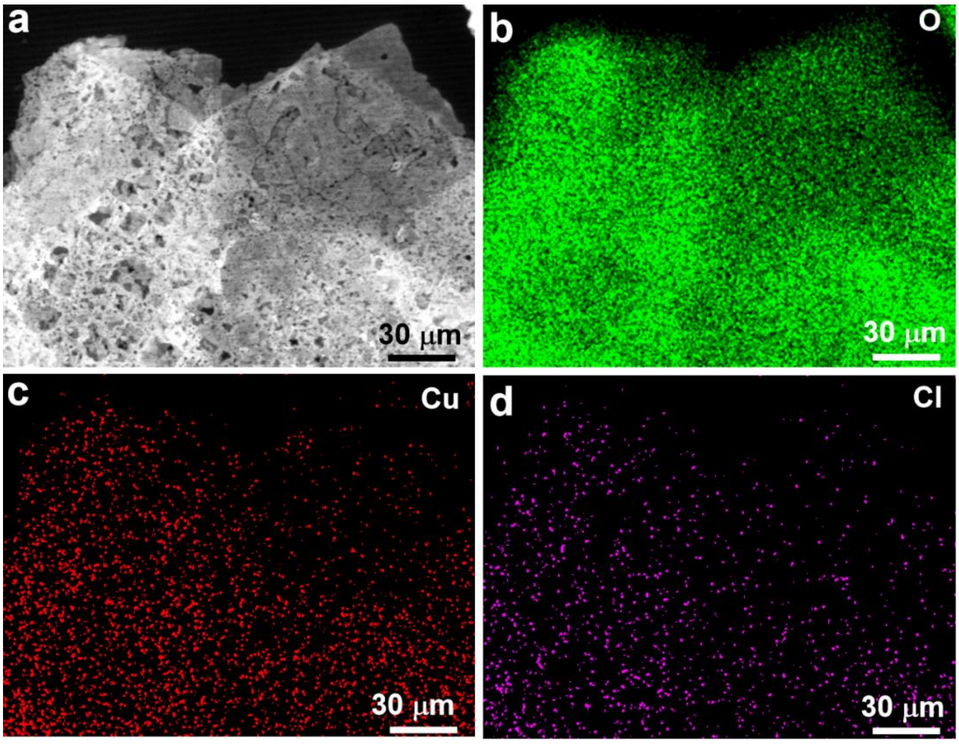

Supplementary Figure 16. EDS mapping images of the O/W microemulsions. (a) The area of EDS mapping recorded from the frozen microemulsions. (b) EDS mapping of O element. (c) EDS mapping of Cu element. (d) EDS mapping of Cl element. In order to find out whether these microemulsions belong to the configuration of W/O or O/W, we analyzed these EDS data. EDS mapping results show that the O element accounts for the largest proportion (atomic ratio), followed by Cu element, and Cl element is the least. Considering that O is mainly from water, Cu is from catalyst and dissolved in water, and Cl is from $CHCl_3$, the current EDS results give us reason to decide that these microemulsions belong to the configuration of O/W.

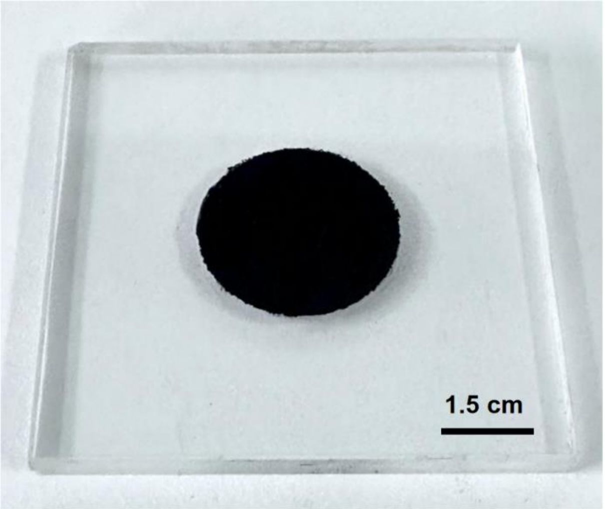

Supplementary Figure 17. The photo of the prepared SERS substrate (2 cm^2) for sensitivity measurement.

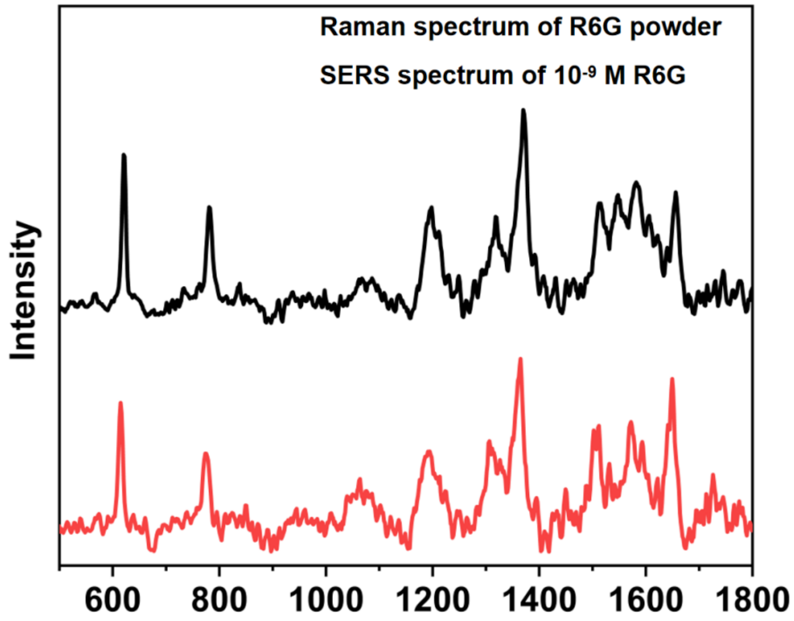

Supplementary Figure 18. SERS spectra of 10^{-9} M R6G on GDY HHMSs and Raman spectrum of R6G powders. SERS spectrum: 532 nm of excitation wavelength, 0.7 mW of laser power, 5 s of integration time. Raman spectrum: 532 nm of excitation wavelength, 0.3 mW of laser power, 1 s of integration time. Source data are provided as a Source Data file.

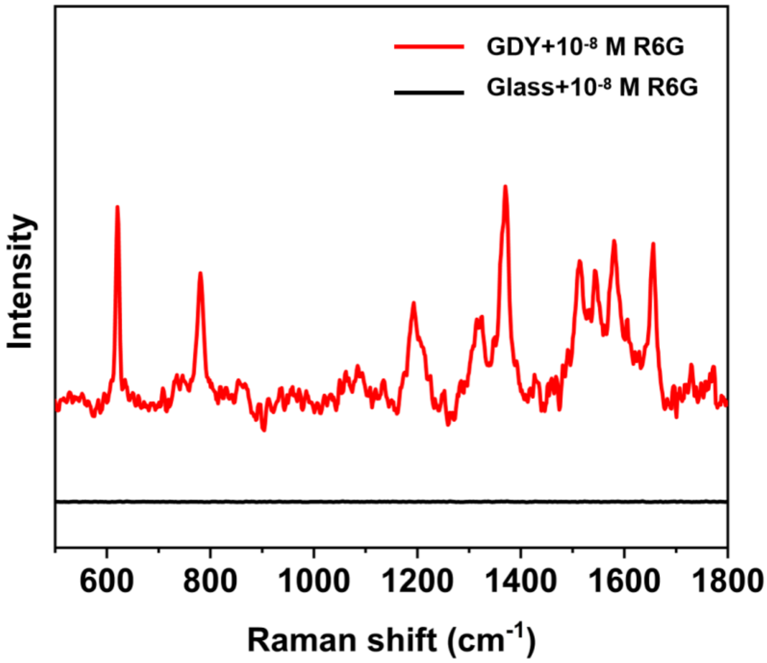

Supplementary Figure 19. Comparison of Raman signals obtained on GDY HHMSs and glass, respectively. Excitation wavelength: 532 nm, laser power: 0.7 mW, integration time: 2 s. Source data are provided as a Source Data file.

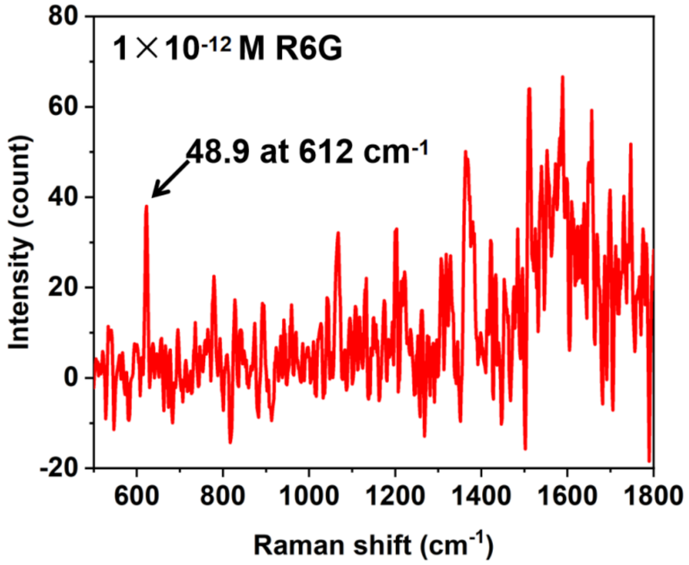

Supplementary Figure 20. SERS spectrum of 1×10^{-12} M R6G obtained on GDY HHMSs. Excitation: 532 nm, integration time: 50 s, laser intensity: 0.7 mW. The measurement contains negative counts is caused by the device's built-in software (Renishaw-inVia Qontor) deducting the fluorescent background. Source data are provided as a Source Data file.

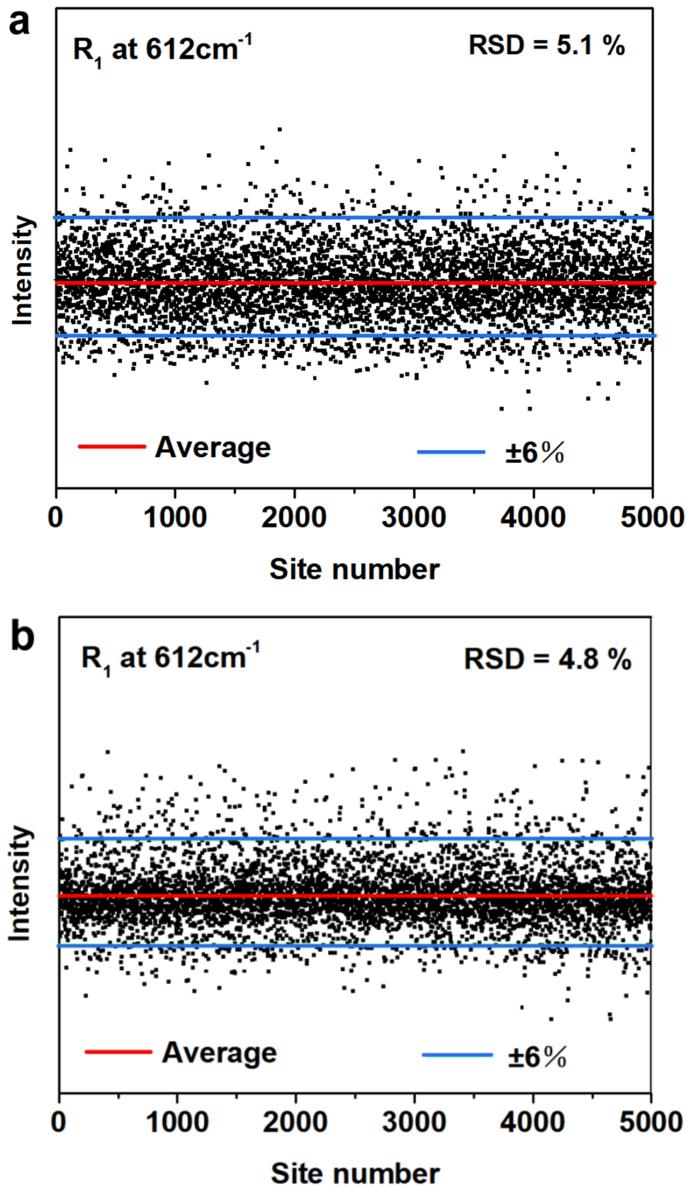

Supplementary Figure 21. (a,b) RSD values of the SERS signal intensities obtained from the other two substrates of the same batch. R6G concentration: 10^{-9} M, excitation wavelength: 532 nm, laser intensity: 0.7 mW, integration time (point): 5 s, scanning area: 2 cm^2. Source data are provided as a Source Data file.

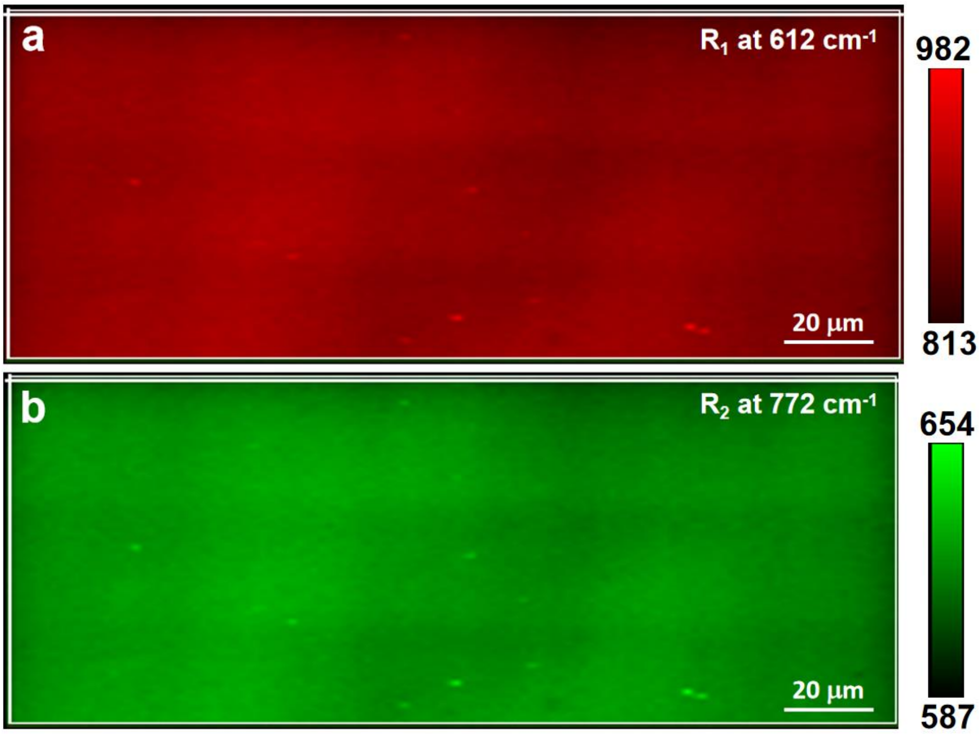

Supplementary Figure 22. SERS mapping images of the GDY/R6G. (a) R_1 at 612 cm^{-1}. (b) R_2 at 772 cm^{-1}. R6G concentration: 10^{-9} M, excitation wavelength: 532 nm, laser intensity: 0.7 mW, integration time (point): 5 s.

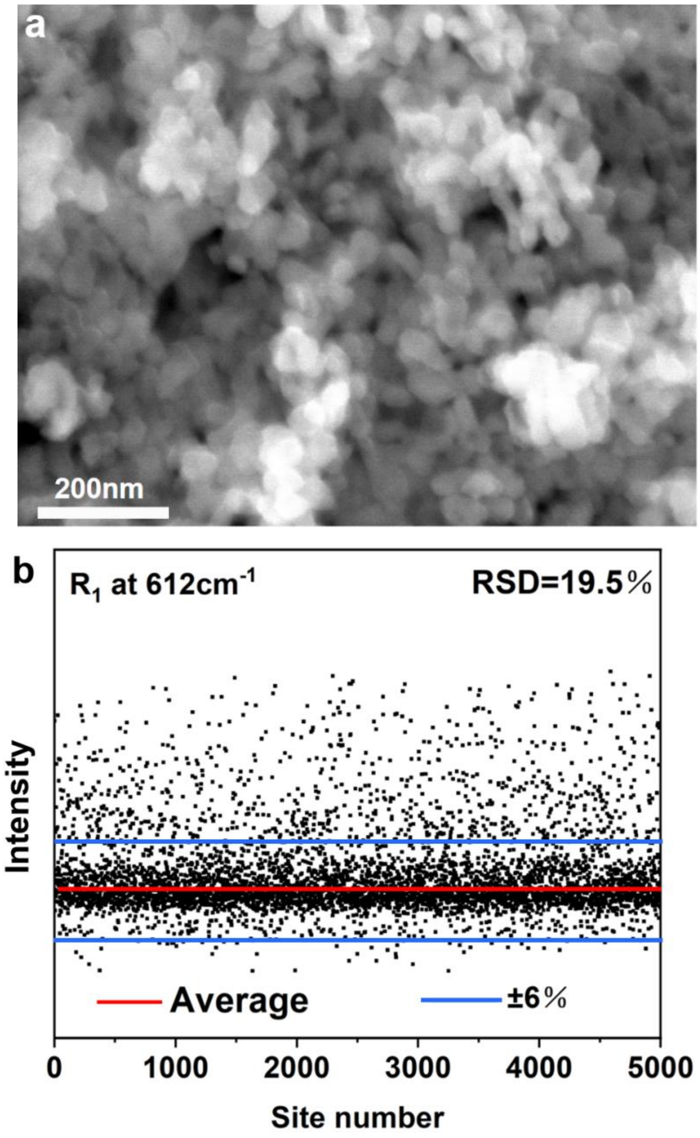

Supplementary Figure 23. RSD of commercial Au nanoparticle SERS substrate. (a) SEM image of the commercial Au nanoparticle SERS substrate. (b) SERS signal intensities at 612 cm^{-1} of 10^{-6} M R6G in a range of 1 cm^2 Au substrate. R6G concentration: 10^{-9} M, excitation wavelength: 532 nm, laser intensity: 0.7 mW, integration time (point): 1 s, scanning area: 2 cm^2. These commercial Au nanoparticles for experiments were purchased from XFNANO company. Source data are provided as a Source Data file.

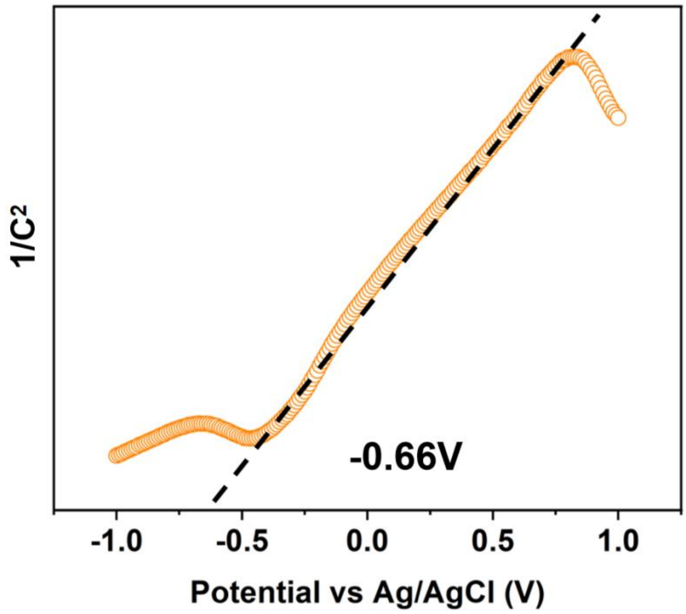

Supplementary Figure 24. Mott-Schottky plots of GDY HHMSs. Source data are provided as a Source Data file.

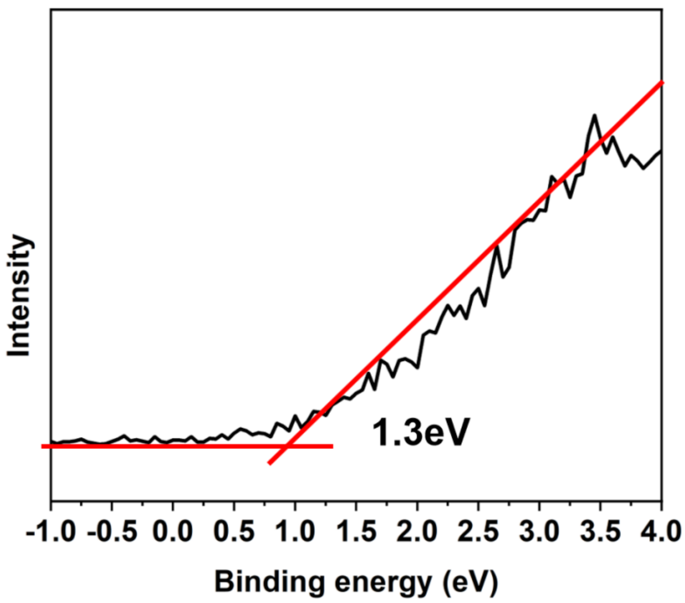

Supplementary Figure 25. XPS valence spectra of GDY HHMSs. Source data are provided as a Source Data file.

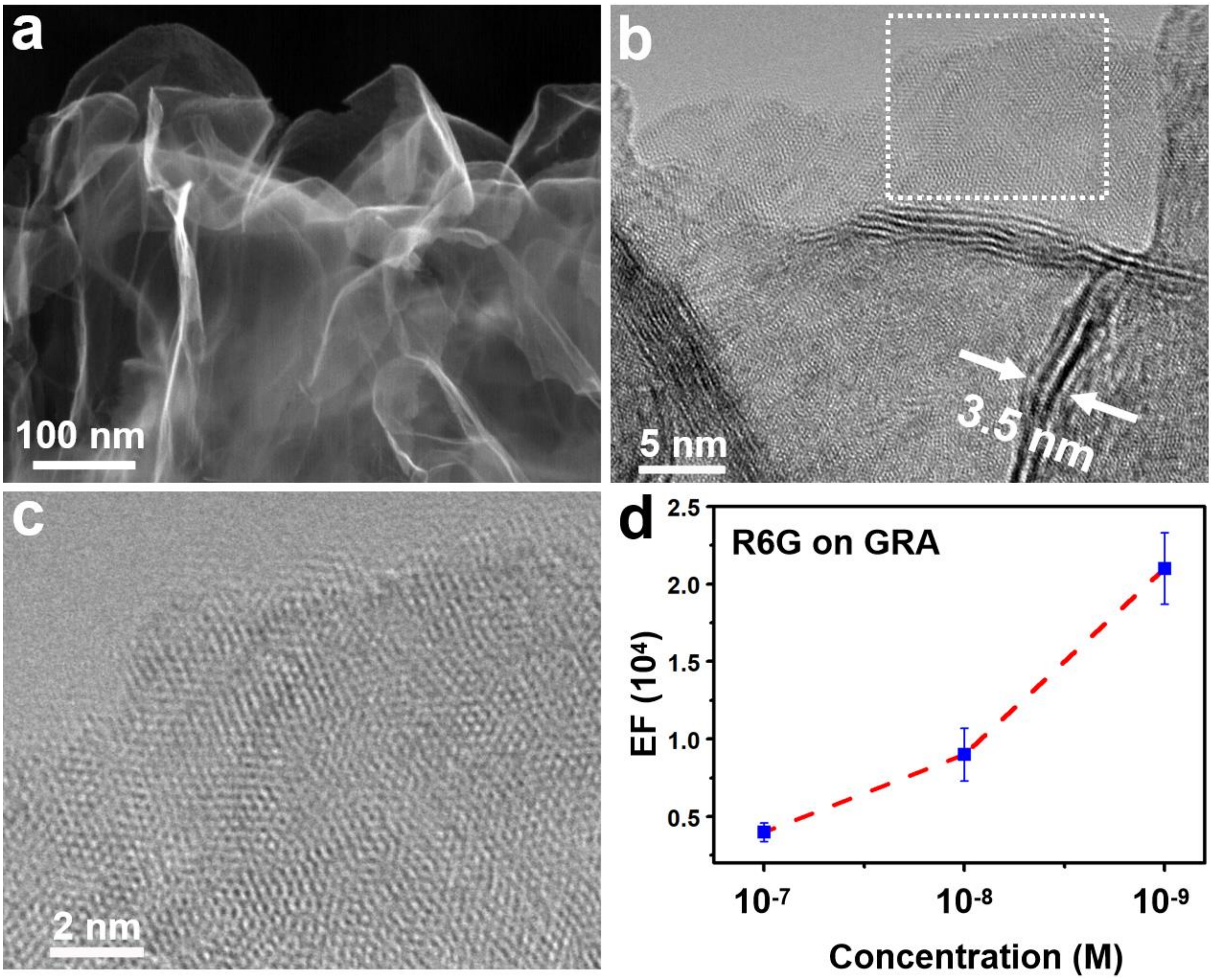

Supplementary Figure 26. Structure characterization and Raman EFs of the graphene. (a) SEM image of graphene nanosheets. (b) HRTEM image of graphene (side view), which shows that its thickness is only about 3.5 nm. (c) The enlarged HRTEM image (vertical view) of the white box area shown in (b). (d) Raman EFs with R_1 (612 cm^{-1} of R6G) at different concentrations. The Error bars are based on the standard deviations of 10 measurements at each concentration. Source data are provided as a Source Data file. These characterizations show that these graphene nanosheets have almost the same thickness as the currently prepared GDY and have high crystallinity. At the same time, the experimental results also show that that at its limit detection concentration (1×10^{-9} M), its maximal Raman EF is 2.1×10^{4} M. These graphene nanosheets for experiments were purchased from XFNANO company, which is a kind of high crystalline graphene ultrathin nanosheets.

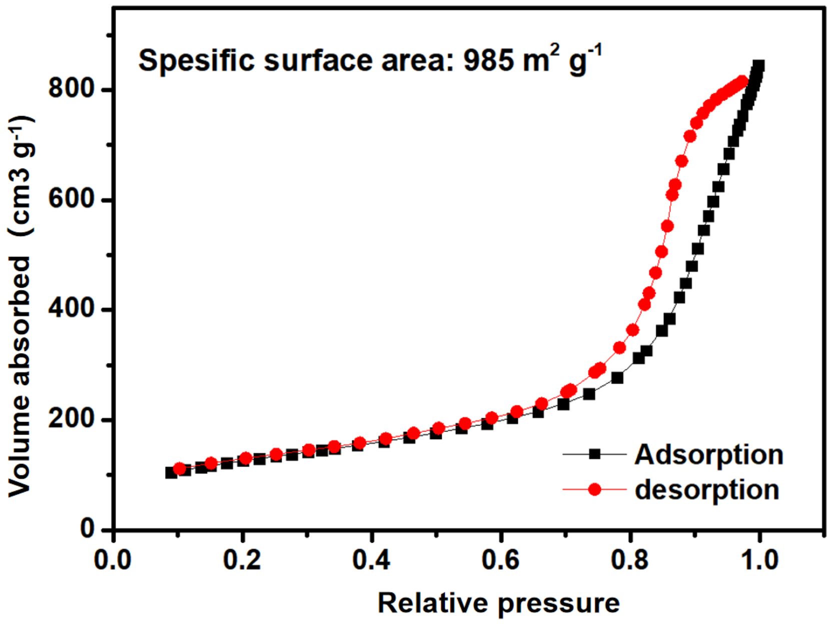

Supplementary Figure 27. N_2 adsorption and desorption isotherms of GRA nanosheets, which shows that its specific surface area is 985 $m^2\ g^{-1}$. Source data are provided as a Source Data file.

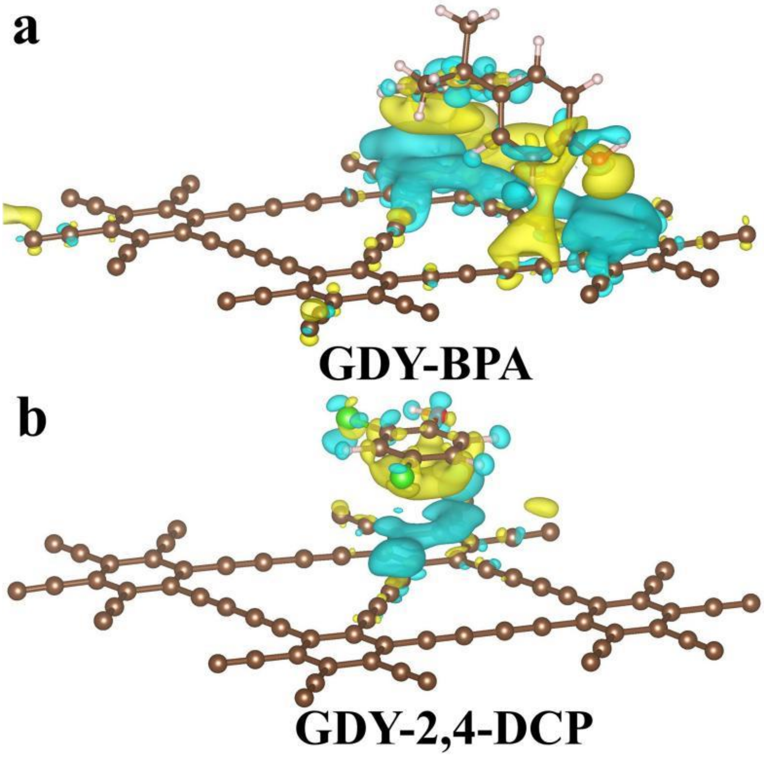

Supplementary Figure 28. Charge density difference of (a) BPA and (b) 2,4-DCP adsorbing on GDY, where the yellow and cyan color mark the region of charge accumulation and depletion, respectively, the isosurface was set to 0.00015 e Å^{-3}.

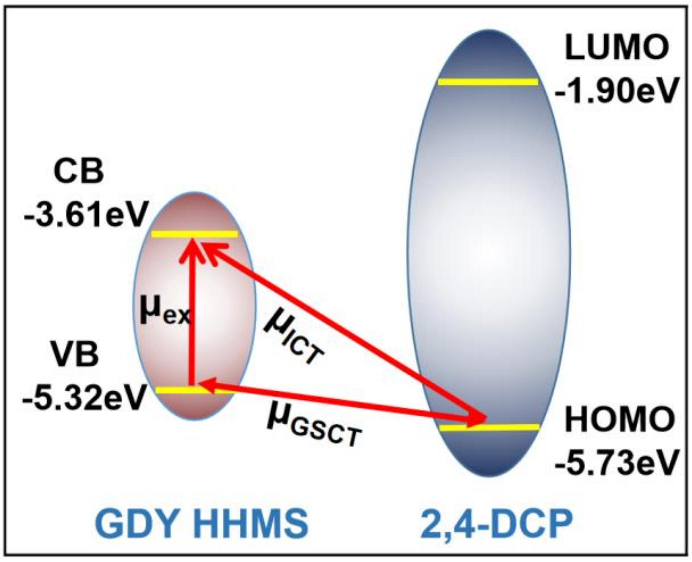

Supplementary Figure 29. Band energy alignment diagram of the charge-transfer pathways in GDY-2,4-DCP system.

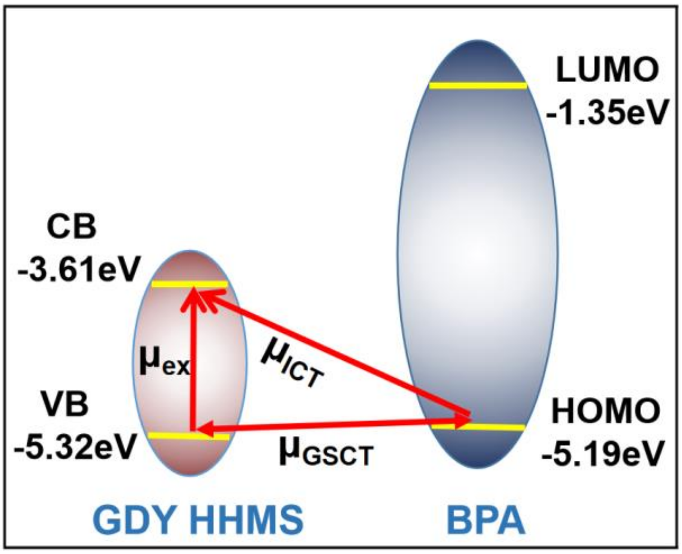

Supplementary Figure 30. Band energy alignment diagram of the charge-transfer pathways in GDY-BPA system.

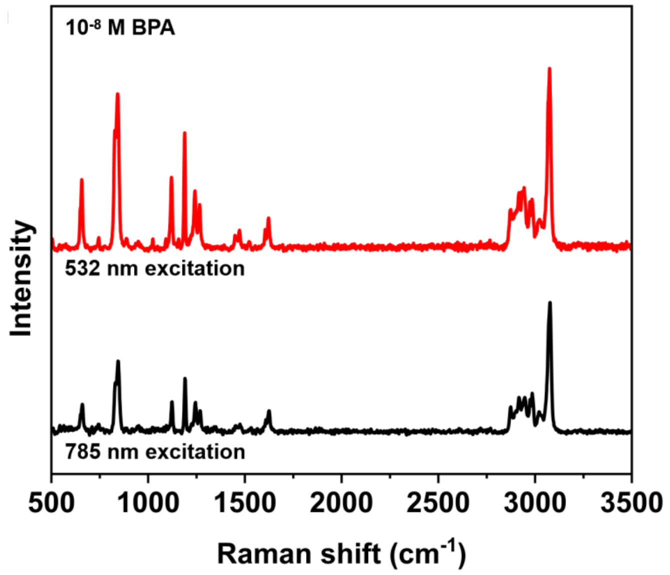

Supplementary Figure 31. SERS spectrum comparison of BPA under different excitation (532 nm and 785 nm). BPA concentration: 10^{-8} M, excitation wavelength: 532 nm and 785 nm, laser intensity: 0.7 mW, integration time: 2 s. Source data are provided as a Source Data file.

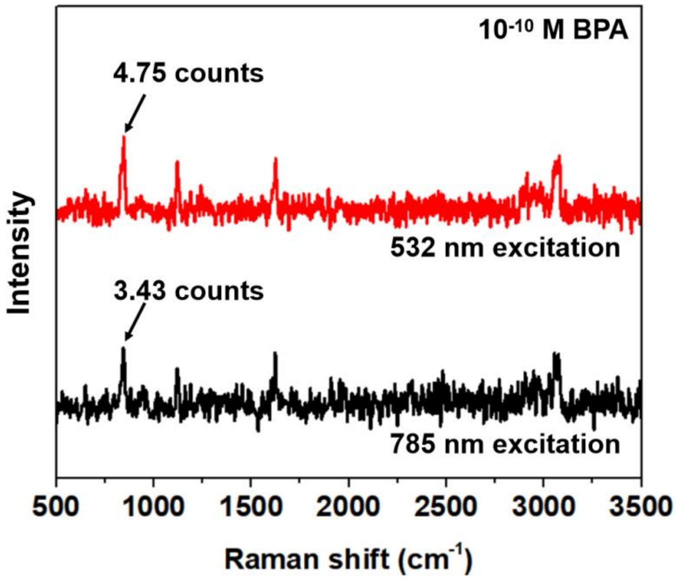

Supplementary Figure 32. SERS spectra of BPA under 532 nm and 785 nm excitation. BPA concentration: 1×10^{-10} M, laser power: 0.7 mW, integration time: 40 s. Source data are provided as a Source Data file.

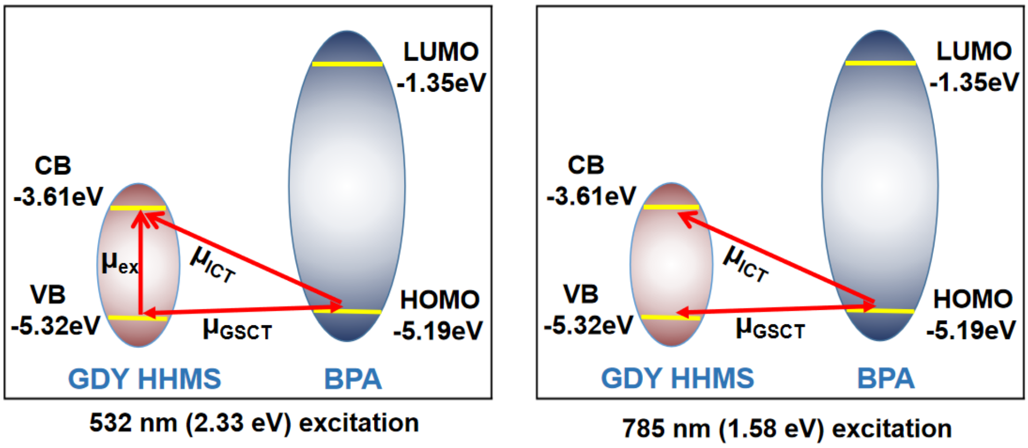

Supplementary Figure 33. Band energy alignment diagram of the charge-transfer pathways in BPA/GDY HHMSs under different excitation.

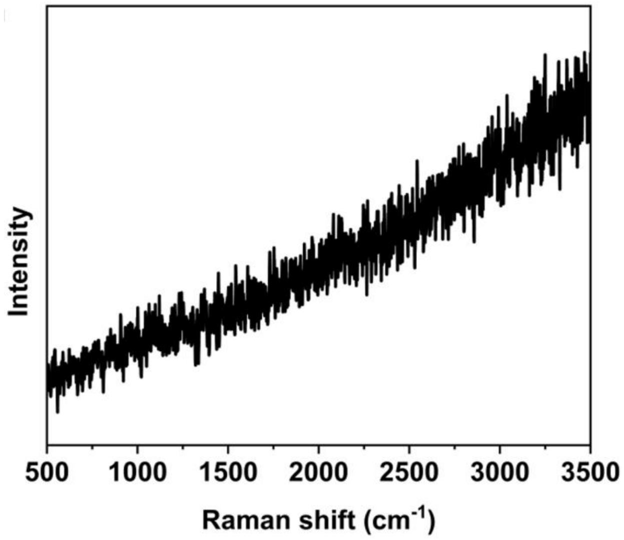

Supplementary Figure 34. SERS spectrum of 2,4-DCP under 785 nm excitation. 2,4-DCP concentration: 1×10^{-10} M, laser power: 0.7 mW, integration time: 40 s. Source data are provided as a Source Data file.

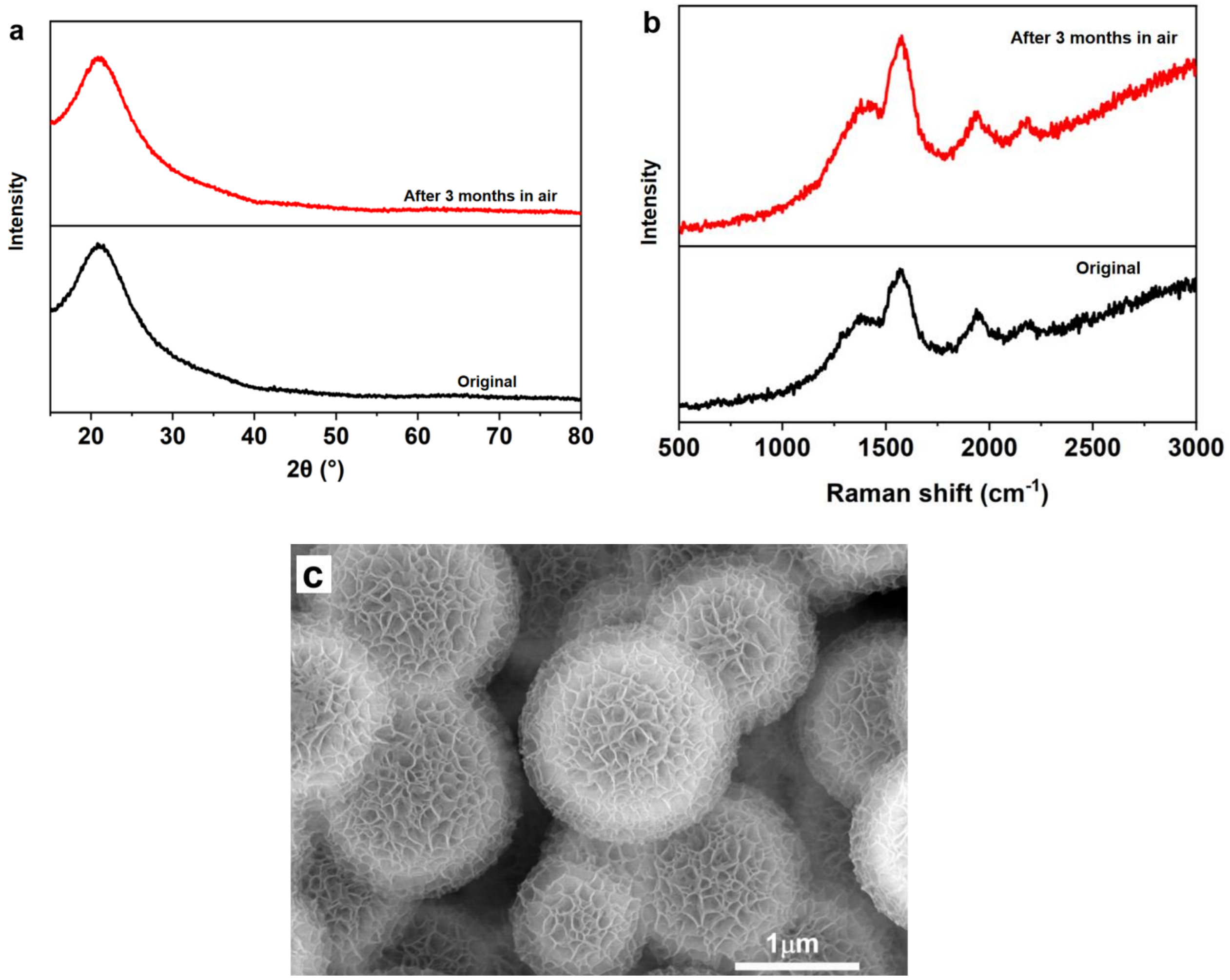

Supplementary Figure 35. Characterizations of Chemical Stability of GDY HHMSs. (a) XRD pattern, (b) Raman spectrum (laser power: 0.5 mW, integration time: 1 s), and (c) SEM image of the GDY HHMSs after 3 months in air. These characterization results demonstrate that the structure and morphology of GDY HHMSs have not undergone significant changes, indicating their high chemical stability. Source data are provided as a Source Data file.

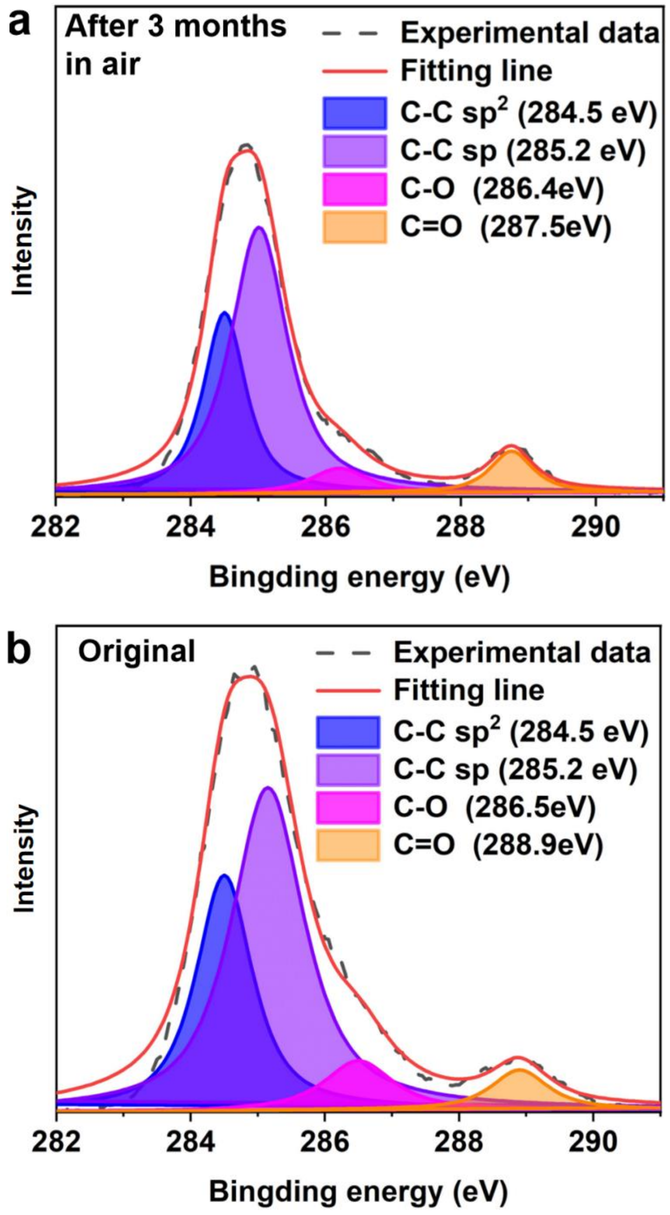

Supplementary Figure 36. (a,b) XPS spectra of the GDY HHMSs after 3 months in air. These results further demonstrate that the structure of GDY HHMSs have not undergone significant changes. Source data are provided as a Source Data file.

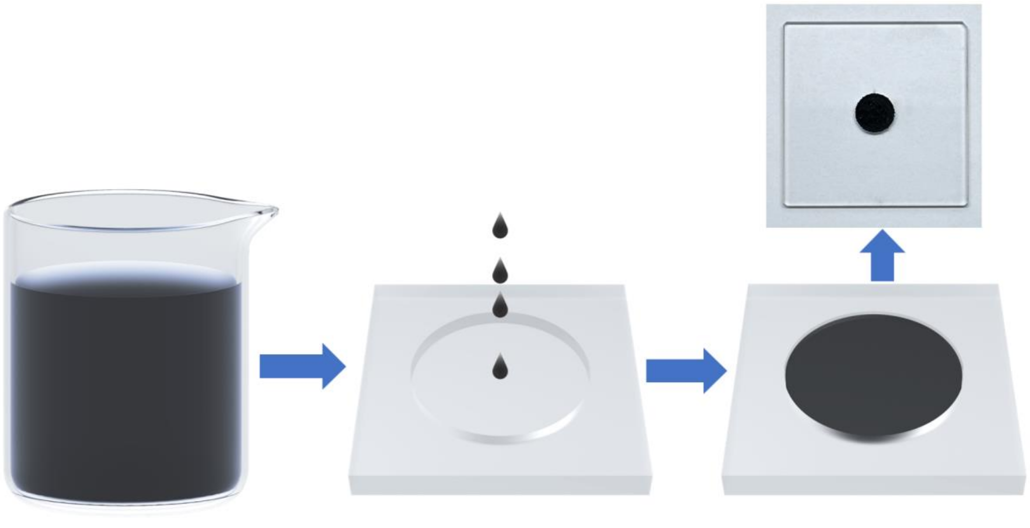

Supplementary Figure 37. SERS substrate preparation diagram and prepared substrate photos for EF calculation.
